# On Wasting or “Simple Atrophy” as It Occurs in Young Children from Insufficient Nourishment

**Published:** 1880-12

**Authors:** Louis Starr

**Affiliations:** Physician to the Episcopal Hospital, and Assistant Physician to the Children’s Hospital, Philadelphia


### ON WASTING OR "SIMPLE ATROPHY" AS IT OCCURS IN YOUNG CHILDREN FROM INSUFFICIENT NOURISHMENT. By LOUIS STARR, M.D., Physician to the Episcopal Hospital, and Assistant Physician to the Children's Hospital, Philadelphia.

Cases illustrating the ill effects of an insufficient supply of food, or of food which, though abundant, is unsuitable in quality, are of frequent occurrence among the out-patients of the Children's Hospital. The condition produced is either one of simple wasting, or of wasting combined with symptoms denoting irritation of the gastro-intestinal canal.

Simple wasting is, I think, most frequently seen in children who have been nursed at the breasts of feeble or overworked mothers, in whom the milk is often both scanty and of poor quality. The symptoms are sufficiently characteristic. There is a gradual loss of plumpness, the muscles grow flaccid, and there seems to be an arrest of growth. The face is pale, the lips pale and thin, the skin harsh and dry or too moist, and the anterior fontanel level or slightly depressed. The temper is usually irritable. When nursed, the child first seizes the nipple ravenously; then, if there is little milk, he quickly drops it, to cry passionately, as if disappointed at not being able to satisfy his hunger; but if the milk is abundant, though thin, he will lie a long time quietly at the breast. The bowels are inclined to constipation. The physical signs connected with the chest and abdomen are negative, and no indication of disease of any special organ of the body can be detected.

Wasting associated with symptoms of gastro-intestinal irritation is more common, and is met with chiefly in infants who are hand-fed. Resulting in the main from an improper diet, it is often encountered where farinaceous food is employed to the exclusion, in great part, of milk; where an infant is allowed or encouraged to bolt bits of table-food and drink tea, and particularly, so far as my experience goes, where the variety of feeding-bottle lately become so popular is used. These bottles have, in place of a plain gum nipple, an arrangement of fine glass and rubber tubing: the glass tubing extending quite to the bottom of bottle, the necessity of holding the latter and keeping it at a proper angle in feeding is avoided. This seeming advantage is counterbalanced by the minor disadvantage that the child, left to itself, is apt to continue suction long after the bottle is exhausted, and by the great disadvantage that the tubing can never be kept clean. During my last three terms of service at the Children's Hospital, it has been my rule to ask for the bottle of every hand-fed infant presented for treatment, and scarcely a day has passed without my seeing several of the bottles referred to. In almost every instance, notwithstanding the most careful and frequent cleansing, the contained milk had a sour odor, and was filled with small curds, while in cases of carelessness the odor was intolerable and the interior of the tubing was encrusted with a layer of altered curd. In bottles provided with a simple nipple, on the contrary, the milk was nearly always perfectly sound and the nipple itself clean.

As there is little difficulty in keeping the bottles themselves clean, there can be only one reason for this difference, namely, in the old-fashioned instrument the nipple is easily removed and as readily inverted and thoroughly cleansed, but in the other there is no way of thoroughly cleaning the twelve or more inches of fine tubing; it cannot be inverted, and the passage of a stream of water or of a small stiff brush can only imperfectly remove the milk clinging to the interior; this, of course, soon undergoes decomposition, and quickly inaugurates change in the next charge of milk placed in the bottle. It is evident that a constant supply of milk thus rendered acid and partially curdled must, like an excess of farinaceous or other unsuitable food, produce irritation of the mucous membrane of the alimentary canal, interfere with the processes of nutrition, and lead to a state in which the features of wasting and disordered digestion are combined.

The following case is a very typical example of this condition :

James ——, æt. three weeks, a foster-child, was brought to the dispensary of the Children's Hospital on April 1, 1879. His nurse stated that he had been in her charge for two weeks, and that he had been sick the whole of this time. The symptoms which arrested her attention were gradual wasting, great restlessness, with frequent prolonged paroxysms of screaming, and redness and excoriation of the skin in the neighborhood of the genitals and anus. He had been fed upon milk diluted with water, the food being given from a nursing-bottle fitted with the combination of glass and rubber tubing.

At the date of application the child was puny, his surface generally was pale and moist, the muscles were flabby, and there was severe intertrigo of the scrotum, groins, perineum, and inner side of each thigh. His mouth felt hot, the mucous membrane was redder than normal, and the tongue and palate were covered with small patches of thrush. He took his food almost ravenously. There were frequent eructations of sour-smelling, partially-coagulated milk, and the bowels were somewhat constipated. Paroxysms of screaming occurred at short intervals, and greatly disturbed his rest at night; during these paroxysms the legs were drawn upward and moved about uneasily, the feet were very cold, and the abdomen was distended and hard. The heart and lungs were healthy, and the urine was voided freely.

The nursing-bottle was examined. The glass tube which extended to the bottom of the bottle was lined with curd, and a quantity of milk remaining from a supply placed in the bottle about an hour before was sour and contained numerous small curds. This change, it was stated, often occurred, in spite of much care taken to keep the bottle and tubing clean.

Directions were given to substitute a soft india-rubber nipple for the tubing, to keep both the bottle and nipple thoroughly clean, to wash out the child's mouth with cold water after each feeding, and to use a food composed of one part of barley-water to two of milk, with the addition of a tablespoonful of lime-water to each half-pint. Small doses of bicarbonate of sodium, with peppermint-water, were prescribed every three hours. The nurse was also ordered to rub half a teaspoonful of warm olive oil into the skin of the abdomen twice daily, to anoint the surface involved in the intertrigo with oxide of zinc ointment, and to keep the feet warm by frictions with the hand.

The improvement under this treatment was rapid. On April 11 (the day of the last visit) his mouth was cool and free from thrush; there was little eructation; the bowels were natural; there were no more attacks of colic; the sleep was undisturbed; the child had begun to gain weight; and the intertrigo was very much better.

In other cases the symptoms are much more grave. The emaciation progresses to an extreme degree; the skin becomes dry, yellowish and harsh, and hangs in loose folds over the bones—and this, although a large quantity of food, such as it is, may be consumed. The combination of great wasting with a voracious appetite is very striking, and is only apparently contradictory, since hunger—the demand of the tissues for reparative material—cannot be appeased by food which, from its bad quality, is incapable of digestion or proper preparation for absorption and assimilation; unsuitable food, too, by irritating the mucous membrane of the stomach, creates a fictitious appetite.

Wasting children sometimes have what are termed "inward spasms." When these spasms occur, the upper lip becomes livid, somewhat everted, and tremulous, the eyeballs rotate or there is a slight squint, and the fingers and toes are strongly flexed. They frequently usher in true convulsions.

These "inward spasms," together with restlessness and irritability of temper, are ordinarily the only indications of involvment of the nervous system; but in a child who recently came under my notice there was a train of very misleading nervous symptoms. The notes are as follows:

Philip ——, 15 months old, of Irish parentage, became an out-patient at the Children's Hospital on April 9, 1880. He was the youngest of five children, all of whom are living and healthy. His father had suffered greatly from rheumatism, and his mother, who brought him to the hospital, looked pale and worn, though she stated that she never felt ill. The child had always been fed at the breast, dentition had progressed regularly, and his health had been good until five days before the date of application, when it was observed that he was more pale than usual, that his movements were more feeble, and that there was a tendency to retraction of the head.

When first seen, his skin was pale, the temperature to the hand was normal, and upon drawing the finger-nail over the surface the tache cérébrale could be faintly produced. His face was listless in expression, the veins of the forehead were distended, and there was marked and rigid retraction of the head, any attempt to move it forward causing cries of pain and being resisted by the contracted cervical muscles. The muscles generally were soft and relaxed. The anterior fontanel was open and was natural in appearance. The tongue was very lightly frosted; there was no indication of pressure upon the gums from an approaching tooth; he took the breast greedily, and his bowels were moved once or twice daily, the stools being perfectly healthy. There was no alteration in the condition of the abdomen. On physical examination the lungs and heart were found to be healthy, though there was a trifling loose cough; the pulse was regular, and slow considering the age of the child, the beats ranging between 80 and 90 a minute. While resting in his cradle, he took little notice, so his mother stated, of what passed in the room about him, but occasionally moved his head restlessly on the pillow and uttered several low, fretful moans. Any attempt at movement, as lifting him up to nurse, was attended by crying.

He was ordered two grains of bromide of potassium, with half a grain of iodide of potassium, in camphor-water four times daily.

On April 12, being too ill to be brought to the hospital, I visited him at his home. I found him in a moderately clean room, in a healthy quarter of the city. He rested passively in his cradle, his head bent backward, his eyelids half closed, his thighs drawn up towards his belly, his arms bent at the elbows, and his hands held up under his chin. There was evidently greater prostration, more wasting, and a nearer approach to stupor. The skin was pale and cool; there was no eruption or maculation; the eyes were sunken and the fontanel somewhat depressed. The breathing was regular, and the pulse was slow and regular, but compressible. The belly was natural in shape, and soft; the tongue was slightly coated; the bowels were moved two or three times daily; there was no vomiting, and the appetite was good. In order to test his capacity for feeding, his mother was directed to put him to the breast; the child began to whimper as soon as he was moved, but when the nipple touched his lips he quickly seized it, made several forcible efforts at suction, and then dropped it with a cry as of disappointment. The breasts were examined and found to be small and flabby, and to contain little or no milk. This discovery suggesting an alteration in the diet, directions were given to feed the child chiefly upon cow's milk, and to reserve the breast-milk for the night. An attempt was also made to increase the flow of the latter by advising an improvement in the mother's dietary. The bromide of potassium mixture was continued.

On April 14 the symptoms were still more serious. When closely questioned, the mother reluctantly admitted that she had not fed her baby as ordered, having no means of procuring milk, and further that, her husband having been out of work for several weeks, her own food had been reduced to a very moderate allowance of bread and tea. In consequence the flow of milk had gradually failed, particularly so during the preceding two days, the knowledge of her child's wants and her total inability to supply them producing an exceedingly nervous, despondent condition, and increasing the effect of the starvation diet.

She was readily put in the way of obtaining substantial relief, and after some trouble reassured and persuaded to persevere in the care of her baby. Improvement began simultaneously with the administration of food; the partial stupor, the retraction of the head, and the depression of the fontanel soon disappeared; the little patient began to gain flesh and color, and his muscles became more firm. The bromide of potassium was stopped, and two drops of the syrup of the iodide of iron were given three times daily.

On April 30, the date of the last visit to the hospital, convalescence was thoroughly established. On September 21 the child was in good health.

In this case the retraction of the head, the boring of the head into the pillow, the peculiar decubitus, the general hyperæsthesia, the semi-stupor, the marked prostration, the slowness of the pulse, the tache cérébrale, and the vomiting suggested the existence of tubercular meningitis; but this opinion was reserved on account of the absence of many characteristics of the disease. Thus, the open fontanel was level; the belly was natural in shape; the bowels were the reverse of constipated; the respiration and pulse were regular; and there was no hydrencephalic cry. Furthermore, no account could be obtained of an initial stage of slowly-failing health, and in other respects the course of the illness was different from that of the suspected disease. At the same time, the idea of such symptoms being due to partial starvation was not entertained until they began to disappear almost immediately upon an increase in the quantity of food. Taking a retrospective view, however, it is easy to refer the anomalous symptoms to an intensely excitable nervous system—a condition depending upon insufficient nourishment, and differing merely in degree from that leading to the "inward spasms" already referred to.

In a paper on "Starvation Fever," recently read by Dr. Da Costa before the College of Physicians of Philadelphia, three cases are detailed in which a still different set of symptoms were noted.

The first case, a girl aged three years, had frequent vomiting, great weakness, giddiness, a feeble and rapid pulse, fever, and loss of vision. Death took place on the thirteenth day.

The second, a pallid, badly-nourished boy four months old, had repeated convulsions and a petechial eruption. Death occurred at the end of twenty-four hours.

A post-mortem examination was made in both instances, and, besides minor alterations, the gastro-intestinal mucous membrane was found to be pale and thin, and there was an effusion of serum into the pleural sacs.

The third patient, a feeble girl of three and a half years, had fever, followed in a short time by bronchitis, which subsequently ran into broncho-pneumonia. Recovery took place on quinine and a upporting treatment. The bronchitis and broncho-pneumonia undoubtedly depended only indirectly on the starvation, which, by leading to weakness and ill health, rendered the child very susceptible to the ordinary causes of catarrh.

In the treatment of wasting from insufficient nourishment the first thing to be attended to is the diet. Without entering at length into this subject, it may be stated, as a general rule, that in selecting a diet the object should be to fix upon one which is suited to the age and digestive powers of the child, so that he may be able to digest, and therefore be nourished by, all the food consumed.

At the hospital I have found that children under twelve months, who have to be either partially or entirely "brought up by hand," ordinarily do well upon cows' milk with lime-water or with barley-water. The food should be administered from a bottle capable of holding half a pint, made of colorless glass, so that the least particle of dirt may be readily seen, and provided with a soft india-rubber nipple. The whole quantity of food intended to be given in a day should never be prepared at once, but each portion should be made freshly and separately at the time of administration. Thus, a bottle such as described, as nearly absolutely clean as possible, may be filled with a mixture of one part of lime-water to two or three of sound milk, or with one part of barley-water to two or three of milk, to which may be added from one to four teaspoonfuls of cream and one or two lumps of cut loaf-sugar ; the nipple (also perfectly clean) is next applied, and the bottle placed in hot water until the contents become warm, when it is ready for the child.

The degree of dilution of the milk and the proportion of cream added vary, of course, with the age. Lime-water is used as the diluent when there is frequent vomiting or acid eructation, barley-water* when it is desired to prevent the formation of a large compact curd.

After the digestion has been brought into good condition by such a diet, the food may be cautiously increased to the point

* Barley-water is made by putting two teaspoonfuls of washed pearl-barley in a pint of water, boiling down to two-thirds of a pint, and straining.

suitable for a healthy child of the same age: for instance, at eight months from two to four fluid ounces of thin mutt^n- or chicken-broth, free from grease, may be allowed in addition to the milk; at twelve months the yelk of a soft-boiled egg, rice and milk, and carefully-mashed potatoes; and after sixteen months a small quantity of finely-minced meat.

Once daily the patient should be bathed in warm water, or, at least, sponged over with warm water, and every morning and evening a teaspoonful of warm olive oil or of cod-liver oil should be gently rubbed into the skin, especially of the abdomen and chest. At the same time the belly should be completely covered with a soft flannel binder, and the feet kept warm. In this way attacks of colic, if not entirely prevented, are rendered much less frequent and severe.

When there is intertrigo, cleanliness and the free use of oxide of zinc ointment usually suffice to effect a cure.

Of medicines, bicarbonate of sodium, pepsin and cod-liver oil are, perhaps, most useful. Cod-liver oil should not be given until the digestive powers have been brought into a comparatively normal state by proper food, antacids and digestants. It seems to be most easily borne when given in emulsion, and may be advantageously combined with lacto-phosphate of lime or with the hypophosphites.

Such symptoms as constipation and diarrhœa demand, of course, appropriate treatment.—*Phila. Med. Times.*

